# Breast cancer incidence in mobile screening vs. in-hospital screening programmes based on 6 313 607 mammograms in 2 387 756 women in Taiwan

**DOI:** 10.7189/jogh.14.04190

**Published:** 2024-09-13

**Authors:** Vu Pham Thao Vy, Amy Ming-Fang Yen, Melissa Min-Szu Yao, Yeun-Chung Chang, Hsian-He Hsu, Giu-Cheng Hsu, Cindy S Lee, Li-Ju Lin, Shu-Li Chia, Chao-Chun Wu, Wing P Chan

**Affiliations:** 1 International PhD Program of Medicine, Taipei Medical University, Taipei, Taiwan; 2 School of Oral Hygiene, College of Medicine, Taipei Medical University, Taipei, Taiwan; 3 Department of Radiology, Koo Foundation Sun Yat-Sen Cancer Center, Taipei, Taiwan; 4 Department of Radiology, National Taiwan University College of Medicine, Taipei, Taiwan; 5 Department of Medical Imaging, National Taiwan University Cancer Center, Taipei, Taiwan; 6 Department of Radiology, Tri-Service General Hospital and National Defense Medical Center, Taipei, Taiwan; 7 Department of Radiology, Kang-Ning General Hospital, Taipei, Taiwan; 8 Department of Radiology, State University of New York, Stony Brook, New York, USA; 9 Health Promotion Administration, Ministry of Health and Welfare, Taipei, Taiwan; 10 Department of Radiology, Wan Fang Hospital, Taipei Medical University, Taipei, Taiwan; 11 Department of Radiology, School of Medicine, College of Medicine, Taipei Medical University, Taipei, Taiwan

## Abstract

**Background:**

Since 2010, Taiwan has conducted a population-based breast cancer screening programme that includes mobile units to improve the screening rate. This study aimed to compare the results of mobile breast cancer screening units with those of hospital-based units and estimate the preclinical detectable phase (PCDP) transition time for Taiwanese women with breast cancer.

**Methods:**

This retrospective cohort study included women aged 45 to 69 years who participated in the programme from 2010 to 2018, with at least two years of follow-up, allowing time to detect breast cancer and determine mortality status. The five-state Markov exponential regression model was used to find (1) the underlying incidence of breast cancer for each location type and (2) the sensitivity that each mammography type offers for detecting PCDP early- and late-stage asymptomatic breast cancer. These parameters shed light on the natural history of breast cancer.

**Results:**

Between 2010 and 2018, 2 387 756 women were screened via 6 313 607 mammograms, 55% of which were performed in hospital-based units. The annual pre-clinical incidence rate per person was 0.0035 (95% CI = 0.0035–0.0036) in hospital units, greater than that in mobile units (0.0022, 95% CI = 0.0022–0.0022). The progression rate from early to late stage within the PCDP was 0.2950 (95% CI = 0.2877–0.3025). The progression rates to the clinical phase from PCDP with early- and late-stage breast cancer were 0.1762 (95% CI = 0.1713–0.1811) and 0.4157 (95% CI = 0.4056–0.4258), leading to calculated mean sojourn times of 3.50 (95% CI = 3.45–3.56) years and 2.36 (95% CI = 2.30–2.42) years, respectively. Using computed radiography to collect the mammograms during PCDP with early-stage breast cancer resulted in a sensitivity of 0.503 (95% CI = 0.484–0.521), whereas using digital radiography resulted in a sensitivity of 0.669 (95% CI = 0.653–0.684). When used during PCDP with late-stage breast cancer, these sensitivities were 0.629 (95% CI = 0.609–0.648) and 0.797 (95% CI = 0.782–0.811), respectively.

**Conclusions:**

The incidence of breast cancer was greater among women visiting in-hospital screening than mobile screening, and the mammogram type was the primary factor affecting sensitivity for detecting asymptomatic breast cancer.

According to the 2022 Cancer Registration Report of the Ministry of Health and Welfare, breast cancer is the most common cancer among women in Taiwan [1].The Taiwan Health Promotion Administration (HPA) has launched the National Breast Cancer Screening Program and offered free breast cancer screenings biennially to women aged 50 to 69 years since January 2004 and women aged 45 to 69 years since November 2009. In 2020, women aged 40–44 years with second-degree relatives ever diagnosed with breast cancer were included in the screening programme. Initially, mammographic screening services were primarily provided using hospital-based units; since 2010, the program expanded to include mobile van units. Since 2014, the incidence rate of breast cancer in Taiwan has fluctuated, while mortality rates have steadily declined [2].

Mammographic screening increases life expectancy and reduces mortality and treatment morbidity [3–5]. The availability of mobile units offers a convenient alternative for women, reducing obstacles to breast cancer screening and increasing the number of mammographic screenings. Those living in rural areas tend to be diagnosed at later stages because they lack facilities and face difficulties finding transportation to cancer screening centres, which are typically in cities. Providing mammographic screenings using mobile transport units is a good option for such women [6]. In Brazil, a programme using mobile screening units screened 122 634 women, reaching 54.8% of the target population [7]. Additionally, a retrospective study in France indicated that mobile mammography units can reduce social and territorial inequality [8].

The length of the preclinical detectable phase (PCDP) is the most important parameter for evaluating a screening program; this refers to the sojourn time: the period in which a tumour is asymptomatic but detectable using screening tests [9]. When the PCDP is sufficiently long, screening can provide benefits such as reduced mortality due to cancer, reduced number of years lost to cancer, reduced morbidity, and improved quality of life [9,10]. The PCDP is difficult to quantify because it cannot be directly observed. Additionally, each mathematical method used to estimate PCDP produces a different result. Methods used with breast cancer include Markov chain models [11–13], incidence and prevalence estimates [14,15], a Bayesian nonlinear mixed-effects model [16], and Launoy’s formula [17].

Despite the expansion of breast cancer screening programs and the introduction of mobile units in Taiwan, the effectiveness of these mobile units remains controversial[18–20]. Therefore, this study aimed to compare the results obtained by mobile and hospital-based breast cancer screening units and estimate the PCDP for Taiwanese women with breast cancer. We also evaluated the natural history of breast cancer in women who participated in national mammographic screenings from 2010 through 2018, following them until the end of 2020.

## METHODS

The Taipei Medical University Joint Institutional Review Committee approved this study, and patient informed consent was waived because of the retrospective nature of the study (TMU-JIRB No. N202311058). The National Breast Cancer Screening Program is administered by the HPA; it reviews and monitors screening procedures, film interpretation procedures, and data entry procedures. All radiologists involved in the program were trained, and all radiation technologies were qualified.

### Data sources

To verify the diagnosis of breast cancer, we reviewed the electronic screening, referral, and diagnosis records uploaded to the HPA-run surveillance and data monitoring centre and linked to the Taiwan Cancer Registry. During the first period, 2004–2009, the government offered a screening program in hospitals only for women aged 50–69 years. In 2010, the program was expanded to mobile units and included women aged 45–69 years. Qualified radiologists read screening mammograms using the American College of Radiology Breast Imaging Reporting and Data System (ACR BI-RADS) [21].

### Screening setting and types of mammograms

Since 2010, mobile and hospital-based units have provided mammograms using either computed radiography (CR) or digital radiography (DR). The former uses photostimulable phosphor plates that require transfer to a digital scanner for laser scanning to convert the stored image into a digital array. In contrast, the latter uses a solid-state detector or a combination of a light-emitting phosphor and a digital converter, directly generating a digital array and image. Almost all breast cancer screening mammograms are 2D, whereas 3D mammograms are usually used in diagnosis. Between 2010 and 2014, around half the mammograms performed in mobile units used CR, compared to only 10% of those performed in hospital units. Mobile units equipped with either CR or DR units installed in trucks, together with qualified radiographers, provide the community with mammographic screening services. Following the guidelines of the Taiwan Radiological Society, only qualified radiologists are allowed to interpret the images. Qualified radiologists must interpret 1000 mammograms in the past two years, and the corresponding figure for qualified radiographers is 200. Certified radiologists are qualified if they have completed an educational course on mammogram interpretation or quality control and earned 10 training credits annually. To maintain their status as ‘qualified’, radiologists must attend mammography training courses, conducted annually and reviewed every two years, and obtain at least a ‘B’ grade in the mammography review. Institutions must obtain mammography accreditation for facilities and qualified radiologists and radiographers, and any changes in the qualifications of these professionals or the mammography equipment must be reported to the HPA within two weeks.

### Outcome definitions

Between 2010 and 2018, 2 387 756 women became part of breast cancer screening cohorts, and they were followed until the end of 2020 to determine breast cancer status and breast cancer mortality using the link to the Taiwan National Cancer Registry. A woman without breast cancer was considered to be in the ‘normal phase’. Cancer can be detected through screening, and if this occurred when a woman was without symptoms, she was considered to be in the PCDP. If breast cancer was diagnosed while symptomatic, that person was considered to be in the clinical phase (CP). During the pre-clinical phase, breast cancer is asymptomatic but can be identified using screening tests. The current (8th edition) of the American Joint Committee on Cancer system [22] was used to assign the stages of cancer, divided into early stages (phases 0 and 1) and late stages (phases 2 and greater).

### Performance metrics

Using the ACR BI-RADS (5th edition), we evaluated the performance during this period of breast cancer screening. Performance was measured using recall rate, cancer detection rate (CDR), positive predictive value (PPV), sensitivity, and specificity. The recall rate was determined by the proportion of screening examinations assessed as BI-RADS category 0, 3, 4, or 5, among which 0 and 3 require immediate follow-up in one to two months and six months, respectively. The CDR was calculated as the number of screenings detecting breast cancer per 1000 performed. Each screening resulted in one of two outcomes: normal findings, for which another screening mammogram was recommended after two years, and abnormal findings, for which further assessments were required to confirm or exclude malignancy. The 5th edition of the ACR BI-RADS was followed to determine all metrics. From 2010 to 2016, an initial positive interpretation was assigned to BI-RADS categories 0, 4, and 5, and an initial negative interpretation was assigned to BI-RADS categories 1, 2, and 3 without recommendations for immediate follow-up. Starting in 2016, an initial positive interpretation was assigned to BI-RADS categories 0, 3, 4, and 5, among which categories 0 and 3 require additional assessments in 1–2 months and six months, respectively, and categories 4 and 5 require imaging-guided biopsies. Finding ductal carcinoma in situ or invasive cancer at the additional assessment deemed this a true positive result. Any other outcome at additional assessment deemed this a false positive. The additional assessments could involve non-invasive procedures such as additional mammography, ultrasound, or magnetic resonance imaging or invasive procedures such as fine-needle aspiration, core-needle biopsy, or open biopsy. Once a malignant tumour was histologically confirmed, the woman was referred for follow-up to a hospital specialising in cancer treatment. Once referred, these women were not invited for further screening. The PPV was calculated by dividing the number of true positives by the sum of true positive and false positive screenings. To identify interval cancers, the data from the screening program register was combined with information from the hospital-based cancer registry, and those who underwent mammography in the last scheduled screening but did not attend the subsequent screening invitation were contacted. This approach recovered 98% of those who had been lost to follow-up. All the data sources recorded the date of diagnosis, ensuring that all interval cancers met the defined criteria.

### Statistical analysis

**Figure 1:**
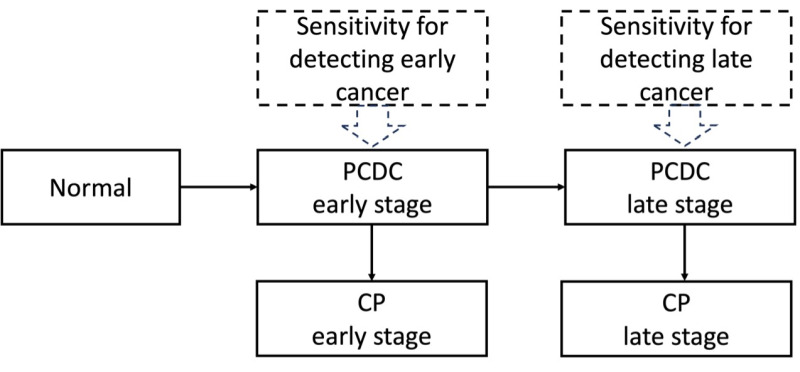
Five-state multistate model of breast cancer progression. CP – clinical phase, PCDP – preclinical detectable phase.

We used the five-state Markov model to estimate the progression of breast cancer. The Markov model has been widely used to describe the dynamic change of states evolving with time or space. In our study using data from mammography screening, our interest focused on the progression of breast cancer before the inception of the disease (normal, State 1) to a PCDP in either early (PCDP early, State 2) or late stages (PCDP late, State 3) and to a clinical phase (CP) in either early (CP early, State 4) or late stages (CP late, State 5). These five states compose the five-state Markov model (**Figure 1**). The instantaneous transition rates from state *i* to state *j*, denoted as λ_ij_, were parameters dominating the progression of the process. Among these, λ_12_ was the incidence of pre-clinical breast cancer per person-year. Others were the instantaneous transition rates for breast cancer. Following the backward Chapman–Kolmogorov equation, the transition rates can be transferred as the transition probabilities in the given observed time intervals [23]. These transition probabilities were the foundation of the total likelihood function. This multi-state Markov process has been well-developed by others to estimate the natural history of diseases [24–27]. Because the characteristics of the women participating in the mobile and hospital screening programs were heterogeneous, the various preclinical incidence rates were recorded in a regression form, and the information describing screening conditions was incorporated into transition rates in an exponential regression form. The Newton-Rapson method was used to optimise the total likelihood function and obtain the maximum likelihood estimate of the preclinical incidence rate, transition rate, and sensitivity. Variance estimates from the reverse Hessian matrix were used to determine the 95% confidence intervals (CIs). All statistical analyses were performed using SAS 9.4, employing the interactive matrix language procedure.

## RESULTS

**Figure 2:**
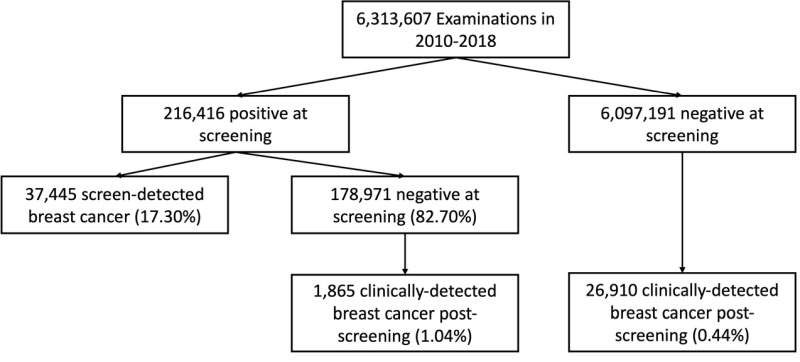
Flowchart of screening and number of diagnosed patients with breast cancer at and after screening.

The breast cancer screening program included 2 387 756 participants, with 6 313 607 screening mammograms collected 2010–2018 (**Figure 2**). In total, 216 416 mammograms (3.43%) showed abnormal or positive results. Of those, 17.30% (37 445) were diagnosed with breast cancer, and by the end of 2018, 1.04% of the remainder (1865 of 178 971) were diagnosed with breast cancer after screening. Breast cancer was clinically detected after 26 910 of the 6 097 191 (0.44%) negative screenings.

The demographics of the study population are shown in **Table 1**. In prevalence screening, the mean age of women undergoing screening mammography was 52.57 ± 6.70 years. The corresponding figure for subsequent screening was 57.69 ± 6.03 years.

**Table 1:** Demographic characteristics for breast cancer screening, 2010–2018

Variables	Prevalence screening, n (%)	Subsequent screening, n (%)
Age group, years (mean + SD, IQR)	52.57 + 6.70, 47–57	57.69 + 6.03, 53–62
*45–49*	1 007 588 (42.20)	405 733 (10.33)
*50–54*	562 268 (23.55)	910 720 (23.20)
*55–59*	375 811 (15.74)	1 045 574 (26.63)
*60–64*	270 965 (11.35)	925 181 (23.57)
*65–69*	171 124 (7.16)	638 643 (16.27)
Breast density		
*Fatty breast*	94 423 (3.95)	183 440 (4.67)
*Scattered fibroglandular density*	509 558 (21.34)	955 338 (24.34)
*Dense breast*	1 326 737 (55.56)	2 161 657 (55.06)
*Extremely dense*	456 990 (19.13)	625 266 (15.92)

IQR -interquartile range, SD – standard deviation

### Key performance indicators: mobile vs. hospital, CR vs. DR, and prevalence vs. subsequent screenings

**Figure 3:**
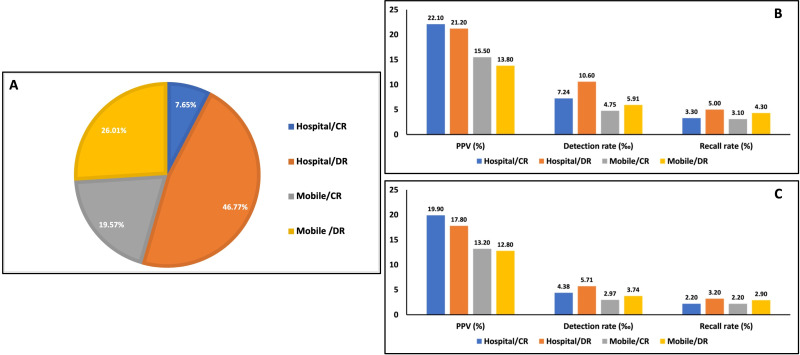
**Panel A.** Proportions of mobile/hospital and CR/DR. **Panel B.** Key performance indicators among mobile/hospital and CR/DR for prevalence screening. **Panel C.** Key performance indicators among mobile/hospital and CR/DR for subsequent screening.CR – computed radiography, DR – digital radiography.

**Figure 4:**
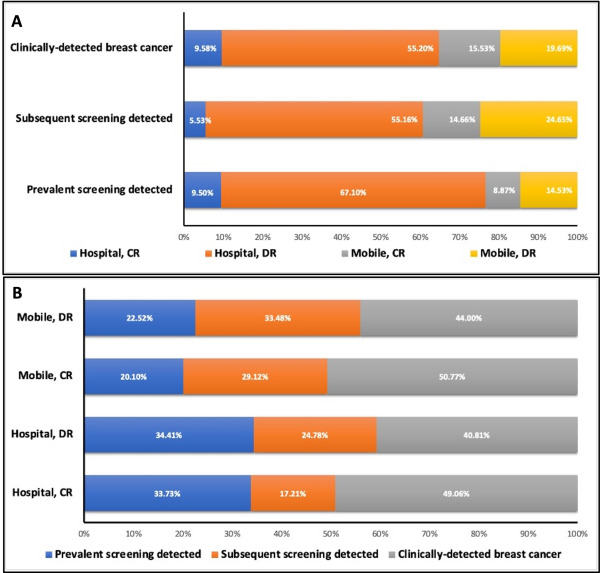
**Panel A.** Distribution of mobile/hospital and CR/DR by detection mode. **Panel B.** Distribution of detection mode by mobile/hospital and CR/DR. CR – computed radiography, DR – digital radiography.

The total screenings performed in the hospital comprised 54.42% (7.65 for CR and 46.77% for DR), and mobile screenings outside comprised 45.58% (19.57 for CR and 26.01% for DR; **Figure 3****,** panel A). The key performance indicators of recall rate, PPV, and CDR are compared between location type and mammogram type at prevalence screening in **Table 2**, **Figure 3**, panel B. The in-hospital screenings performed using DR yielded the greatest recall rate (5.0%) and CDR (10.60 per 1000), and those performed using CR yielded the greatest PPV (22.1%). On the other hand, screenings performed in mobile units using CR yielded the lowest recall and detection rates (3.1 and 4.75%, respectively). **Figure 3**, panel C shows the same trends for subsequent screenings. The greatest recall rate (3.2%) and CDR (5.71%) were also found among the screenings performed in hospital units using DR. Interestingly, the CR mammograms collected in mobile units yielded the same recall rate as those collected in hospitals (2.2%). The majority (15 884 of 28 775, 55.20%) of clinically detected cancers were found after screenings performed in hospital units using DR. Mobile units using DR produced the next-largest number (5665, 19.69%) followed by mobile units using CR (4470, 15.53%) and hospital units using CR (2756, 9.58%; **Figure 4**, panel A). The resultant incidence rates per 1000 person-years were 1.99 and 1.68 for hospital units using CR and DR, respectively, and 1.15 and 1.03 for mobile units using CR and DR, respectively.

**Table 2:** Parameters in screening breast cancer in hospital/mobile units

Unit	Type	Examinations	Recall rate, %	PPV, %	SD	CDR (per 1000)	CD	Person-years	Incidence (per 1000)
		Prevalence screen							
Hospital	CR	261 819	3.3	22.1	1895	7.24			
Hospital	DR	1 262 807	5.0	21.2	13 392	10.60			
Mobile	CR	372 667	3.1	15.5	1770	4.75			
Mobile	DR	490 463	4.3	13.8	2900	5.91			
		Subsequent screen							
Hospital	CR	221 020	2.2	19.9	967	4.38			
Hospital	DR	1 690 021	3.2	17.8	9646	5.71			
Mobile	CR	862 679	2.2	13.2	2564	2.97			
Mobile	DR	1 152 131	2.9	12.8	4311	3.74			
		Post screening							
Hospital							2756	1 381 571	1.99
Hospital							15 884	9 463 708	1.68
Mobile							4470	3 884 579	1.15
Mobile							5665	5 516 319	1.03

CD – clinically detected cancers, CDR – cancer detection rate, CR – computed radiography, DR – digital radiography, PPV – positive predicting value, SD – screen-detected cancers

### Distribution of screening type and detection mode

Prevalence screening resulted in a greater detection rate than subsequent screening, no matter the location of collection or type of mammogram performed (**Table 2**). Of all those screened in hospital units using CR and ultimately diagnosed with breast cancer, 33.73% were detected at the prevalence screening and 17.21% at a subsequent screening (**Figure 4**, panel B). The remainder were clinically diagnosed after screening. Comparatively, when DR was used in a hospital setting, 34.41% of those ultimately diagnosed were detected at the prevalence screening and 24.78% at a subsequent screening. The overall proportions of mobile-to-hospital and CR-to-DR screenings are shown for prevalence and subsequent screenings in **Table 1**. In particular, of all prevalence screenings, 1 262 807 (52.89%) were performed in hospitals using DR, and 261 819 (10.97%) were performed in hospitals using CR. Of all subsequent screenings, 43.04% were performed in hospitals using DR and 5.63% using CR.

### Estimated incidence and progression rate of breast cancer in five-state natural history

**Figure 5:**
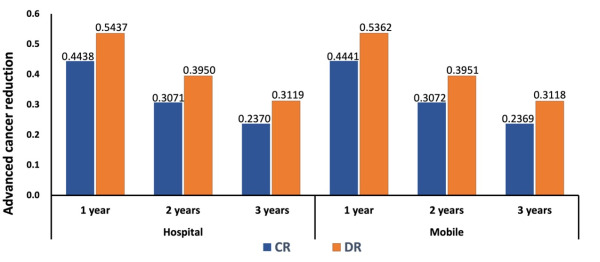
Risk reduction of late-stage cancer by annual, biennial, and triennial screening by CR and DR. CR – computed radiography, DR – digital radiography.

The estimated incidence and progression rates of breast cancer in the five-state natural history are shown in **Table 3**. The annual pre-clinical incidence rate per person for screenings performed in hospitals was 0.0035 (95% CI = 0.0035–0.0036), greater than for those performed in mobile units (0.0022; 95% CI = 0.0022–0.0022). The progression rate from PCDP with early-stage cancer to PCDP with late-stage cancer was 0.2950 (95% CI = 0.2877–0.3025), whereas the progression from PCDP with early-stage cancer to CP with early-stage cancer was 0.1762 (95% CI = 0.1713–0.1811). Comparatively, the progression rate from PCDP with late-stage cancer to CP with late-stage cancer was 0.4157 (95% CI = 0.4056–0.4258). Thus, the mean sojourn time was 3.50 (95% CI = 3.45–3.56) years for early-stage breast cancer and 2.36 (95%CI = 2.30–2.42) years for late-stage breast cancer. The sensitivities for detecting pre-clinical breast cancer in the early stage using CR and DR were 0.503 (95% CI = 0.484–0.521) and 0.669 (95% CI = 0.653–0.684), respectively. Those for detecting late-stage breast cancer were 0.629 (95% CI = 0.609–0.648) and 0.797 (95% CI = 0.782–0.811), respectively. Based on the estimated results of the natural history of breast cancer progression, the risk reductions of advanced cancer via annual, biennial, and triennial screenings performed in hospitals using CR were 0.4438, 0.3071, and 0.2370, respectively. Comparatively, these rates were greater if DR was used (0.5357, 0.3950, and 0.3119, respectively). These figures were 0.4441, 0.3072, and 0.2369, respectively, when mobile units performed CR mammograms and 0.5363, 0.3952, and 0.3118, respectively, when they performed DR mammograms (**Figure 5**).

**Table 3:** Estimated results of incidence and progression rate of breast cancer in five-state natural history, stratified by mobile/hospital and CR/DR

Parameter	Estimate	(95% CI)	
Normal →PCDP early-stage			
*Hospital*	0.0035	0.0035	0.0036
*Mobile*	0.0022	0.0022	0.0022
PCDP early-stage →PCDP late-stage	0.2950	0.2877	0.3025
PCDP early-stage →CP early-stage	0.1762	0.1713	0.1811
PCDP late-stage →CP late-stage	0.4157	0.4056	0.4258
Sensitivity for early-stage cancer			
*CR*	0.530	0.484	0.521
*DR*	0.669	0.653	0.684
Sensitivity for late-stage cancer			
*CR*	0.629	0.609	0.648
*DR*	0.797	0.782	0.811

CI – confidence interval, CP – clinical phase, CR – computed radiography, DR – digital radiography, PCDP – preclinical detectable phase

## DISCUSSION

This study provides insight into the performance of breast cancer screening programmes in Taiwan and highlights the importance of considering factors such as location type and mammography type in evaluating screening effectiveness. Between 2010 and 2018, 2 387 756 women participated at least once in the breast cancer screening programme through mobile and hospital units. We estimate the sojourn time in early-stage cancer to be 3.50 years. Tabar et al. estimated a sojourn time of 3.3 years for women aged 50–59 years in the Swedish Two-County trial [28]. Aarts et al. used a maximum-likelihood estimation, finding the sojourn time to be around 3–4 years when using the Markov Chain Monte Carlo simulation [29]. The shorter follow-up time hindered their detection of slow-growing breast cancer tumours at a younger age. A lower mean sojourn time estimate could result from a shorter follow-up period and ending screening after 69 years of age; this could omit slow-growing breast tumours. A subsequent long-term follow-up study could validate this. In 2010, the screening programme included mobile units and younger women, increasing the total screened population.

From 2010–2018, 6 313 607 mammographic screenings were performed across 2 387 756 women, detecting 37 445 cases of breast cancer. Hospital-based units performed the larger proportion of these screenings (55%). In particular, the largest proportion of the 46.77% was collected in hospitals using DR. The implementation of mobile units provided a convenient option for women, making it easier to participate in the early cancer detection programme. Several authors have mentioned the benefits of using mobile screening units: they lower the barriers to attaining breast cancer screenings, thus increasing the programme reach [18,30,31].

### Key performance indicators: Mobile vs. hospital, CR vs. DR, and prevalence vs. subsequent screenings

CDR was the greatest among prevalence DR mammograms performed in hospital-based units (10.60 per 1000). Fewer cancers were detected by subsequent screenings, no matter the location of collection or type of mammogram performed. Furthermore, CDR was greater among mammograms performed in hospital units compared to mobile units, no matter the mammogram type. We also found that DR performed better in subsequent screenings than in prevalence screenings, and our results exceed those reported in other studies [32–35]. Others have shown that mammographic screenings can detect 2 to 8 cancers per 1000 mammograms. We found that the recall rate was greater among prevalence screenings than subsequent screenings. Our recall rates were lower than those reported for the US (5–12%) but comparable to those reported for Europe (1–4%), where two readers are required to interpret screenings [36-39]. Others have proven that the recall rate is influenced by many important factors, such as patient population, radiologist, and systemic factors [39–41]. We found greater PPVs among screenings collected in hospitals compared to mobile units and in prevalence screenings compared to subsequent screenings. Because those without previous mammograms fell primarily into the prevalence screening group, they would be expected to yield a greater CDR compared to those who underwent subsequent screenings without prior mammograms for comparison [42,43]. One study compared the performance of diagnostic mammograms interpreted with and without the use of prior mammograms and found that having a prior mammogram for comparison led to an increase in CDR in diagnostic mammography but not screening mammography [44]. The authors proposed that small changes in breast tissue over time might be considered more diagnostically suspicious, leading to improved cancer detection. Our study suggests that similar effects can be observed in a screening environment. Certain features seen on mammograms, such as asymmetries, can be seen only as a change compared to previous mammograms and cannot be detected without the comparator.

### Distribution of mammogram type was similar across unit types

Among those visiting mobile units, breast cancer was most often clinically diagnosed followed by detection at subsequent screenings and then detection at prevalence screenings. The trend was similar among those visiting hospital units, except that detection at prevalence screenings is best detected at subsequent screenings. Of those with clinically detectable breast cancer, 74.89% were detected using DR. Identifying those with clinically detected breast cancers is very important because they have a higher risk of carrying rare deleterious mutations in cancer-related genes and often have poorer tumour prognostic characteristics and survival results compared to screening-detected breast cancers [45–47]. Given that CR mammograms perform worse in quantum efficiency and contrast detail analysis compared to DR mammograms [48,49], examining how this category of digital systems affects the performance metrics of screening interventions is essential.

Multi-state models provide a comprehensive understanding of the disease progress and can help estimate the number of individuals in various stages of progression. With the application of a five-state Markov model, the estimated pre-clinical incidence rate for hospital units was found to be greater than that for mobile units. Additionally, PCDP with early-stage cancer will more likely transition to PCDP with late-stage cancer than CP with early-stage cancer.

We used our breast cancer progression results and found that the risk of advanced cancer was reduced by applying annual, biennial, and triennial screenings, no matter the location of collection or type of mammogram performed. Our results show that annual screenings could reduce advanced breast cancers by a large proportion compared to triennial screenings. However, whatever the time frame used for subsequent screenings, the reduction in the rate of advanced breast cancer was greater when DR was employed instead of CR. These findings suggest that women who are annually screened will gain more benefits compared to those who are screened less often. Several others have found similar results [50,51]. Moorman et al. showed that annual screening was associated with diagnosis at an earlier stage compared to biennial or nonannual screening [52]. Bennett et al. showed that in 8289 cases of cancer in women aged 50–64 years, annual screening compared to biennial screening would have resulted in diagnosis at an earlier stage [53]. Tabar et al. suggested that shortening screening intervals could be beneficial in identifying early breast cancer in women aged 40–49 years, given that tumours are likely to progress relatively quickly in this population [28]. Additionally, Carlos et al. indicated that the screening interval has a different effect depending on age – they suggested annual screenings for women aged 50–69 years, biennial screenings for young women, and longer screening intervals for women aged 70–74 years [54]. One study showed that annual screening had a lower recall rate compared to biennial screening [55]; they also found that annual screening led to the identification of smaller tumours, which tend to have more favourable outcomes.

The strengths of the study are the large number of individuals included, the data source (a national screening programme), and the identification of the role of mobile units as well as the thresholds for transition from pre-clinical to clinical phases. This study also has some limitations. First, it was designed as a retrospective study and lacked prospective randomisation. However, the screening programme was conceived from this perspective, and the technical and interpretation qualities have been well-monitored by government regulations. Second, the retrospective design of the study may have introduced selection biases such as time bias. A screening programme does not allow for the detection of all breast cancers in the screened population, some tumours are still identified based on symptoms (interval cancer cases). The short follow-up period might have led to an underestimation of slow-growing tumours because these tumours may not have been detected within the study timeframe. Future studies with a prospective design and longer follow-up periods are necessary to confirm our findings and better understand the long-term outcomes of different screening programmes. Third, due to a lack of information, we could not account for certain factors such as the genetic or epidemiological effects or known confounders associated with the risks of breast cancer.

## CONCLUSIONS

The mean PCDP times of early- and late-stage breast cancers were estimated at 3.50 and 2.71 years, respectively. The estimated pre-clinical incidence rate was greater in hospital-based units compared to mobile units, and the recall and detection rates were greater among DR mammograms collected in hospital-based units. These results highlight the importance of considering social determinants of health and access to screening as an essential component of a national breast cancer screening programme.
